# Medical malpractice in Oman: A 12-year retrospective record review

**DOI:** 10.1371/journal.pone.0290349

**Published:** 2023-08-23

**Authors:** Amal A. AlBalushi, Abdullah Al-Asmi, Waleed Al-Shekaili, Rana Rafiq Kayed, M. Mazharul Islam, Aishwarya Ganesh, Samir Al-Adawi

**Affiliations:** 1 Higher Medical Committee, Muscat, Oman; 2 Neurology Unit, Department of Medicine, College of Medicine and Health Sciences, Sultan Qaboos University Hospital, Muscat, Oman; 3 Head of Health Systems Research, Ministry of Health, Muscat, Oman; 4 Emergency Medicine Specialist / Rapporteur, Higher Medical Committee, Muscat, Oman; 5 Department of Statistics, College of Science, Sultan Qaboos University, Muscat, Oman; 6 Department of Behavioral Medicine, College of Medicine and Health Sciences, Sultan Qaboos University, Muscat, Oman; University of Macerata: Universita degli Studi di Macerata, ITALY

## Abstract

**Background:**

There is a paucity of studies documenting medical malpractice litigation in countries of the Arabian Gulf, such as Oman.

**Objectives:**

To describe the characteristics of malpractice claims, the outcomes decided by the medical liability committee, and predictors of medical errors.

**Methods:**

This is a retrospective observational study that reviewed medical malpractice cases registered in Oman over a 12-year period (2010–2021) with the medical liability committee, known as the Higher Medical Committee (HMC). Descriptive and inferential statistical techniques, including multiple logistic regression techniques, were used for data analysis.

**Results:**

Between 2010 and 2021, the HMC registered 1284 medical malpractice cases, out of which 1048 were fully investigated. The number of registered cases increased during this period. These cases included those raised by Omani nationals and expatriates, with a majority (86%) raised by Omani complainants. Two-thirds (67%) of the cases involved adult complainants aged 18–60 years. About 43% of the cases were from the urban Muscat region, and 68% were related to public hospitals. The most common specialties involved were obstetrics and gynecology (20.1%), internal medicine (19.7%), surgery (17.6%) and orthopedics (13.8%). Half (51%) of the appeals or grievances were dismissed because they were not preceded by medical negligence or malpractice. The average waiting time to initiate the investigation was 10 months. Errors were more common among non-Omani complainants and cases related to private hospitals. Significant predictors of errors included nationality (i.e. Omani vs. non-Omani), the referring institution, the medical specialty and the type of health institution involved, and the waiting time to initiate the investigation.

**Conclusion:**

To date, the number of cases of medical malpractice in Oman is lower compared to international trends, although there has been an upsurge in recent years. More research using a more robust methodology is warranted to contextualise the factors that contribute to this upward trend, as well as the preponderance in urban settings and among certain demographic populations.

## 1. Introduction

Medical malpractice is defined as a deviation from acceptable good standards of medical practice, which takes the form of acts or omissions and results in injury, harm, or death [1, 2]. Medical malpractice is handled differently in different countries. In the United States, malpractice cases are generally civil offenses [1], while in Japan and China, they are criminal offenses [3, 4]. In Italy, most claims are pursued through the civil court system rather than through criminal law [5].

To date, there is a dearth of reports and analysis of malpractice claims or lawsuits in Arabian Gulf countries, where the recent economic growth of the fossil fuel and transshipment industries has resulted in rapidly improved standards of living and an exponential growth of the health industry. A prevalent issue in these countries is a labor shortage in the healthcare sectors, resulting in a dependency on an expatriate workforce [6]. One such Arabian Gulf country is Oman, with an estimated 5 million strong population, of which approximately half are expatriate workers and their families [7]. About 58% of the health workforce is Omani, and the remaining proportion of expatriate healthcare workers immigrate from different parts of the world, thus bringing with them their own cultures about healthcare safety standards and practices [8]. In Oman, the Ministry of Health (MOH) is the principal provider of health care and is responsible for the supervision and coordination of government hospitals, public health centers, and private institutes [9]. Oman has a universal free healthcare system, and most health services are run by the government (i.e. MOH). Oman aspires to fulfil the Sustainable Development Goals of the United Nations, including Goal 3 which aims to ensure healthy lives and promote well-being for all of all ages [10]. The MOH established the Directorate General Quality Assurance Centre (DGQAC) to lead patient safety guidelines, policies and audit activities in Oman’s healthcare institutes, and the DGQAC was designated as a collaborating center by the World Health Organization (WHO) for quality and patient safety training. The MOH also adopted the WHO’s ‘Patient Safety Friendly Hospital Initiative’ (PSFHI) to reduce rates of preventable adverse outcomes in hospital settings [11]. Some healthcare systems in Oman are accredited by international organizations that emphasize accountability in medical practice [12].

All healthcare workers are expected to adhere to established standards of care, clinical guidelines and protocols [13]. A few basic principles associated with patient safety and positive treatment outcomes include obtaining informed consent from patients, effective provider-to-patient communication, and maintaining accurate and comprehensive medical records. A study assessing healthcare professionals’ perceptions regarding patient safety culture in hospitals in Oman found that the safety culture dimensions with the lowest positive scores included ‘non-punitive response to error’, ‘staffing’, and ‘hand-offs and transitions’ [14]. Another study by Al Balushi et al reported that significant factors associated with higher self-reported medical error rates among healthcare professionals included male gender, Omani nationality, younger age, occupational burnout, and exposure to work-related bullying [15].

In the event of a malpractice claim or lawsuit, the Oman Medical Association provides to their members a professional law firm to help manage their defense in the judiciary system. In addition, all government healthcare workers are covered by the national compensation fund for any financial liability which may arise from such medical malpractice claims. Similarly, the MOH mandates that all private healthcare institutions have liability insurance to protect their healthcare workers against malpractice litigation. There is a paucity of studies documenting medical malpractice litigation in the Arabian Gulf countries, and Oman is no exception. Over time, Omani law has established policies related to the regulation of health services and, when its integrity is compromised, the process of handling malpractice. According to the MOH, medical liabilities that healthcare professionals may be associated with include (i) penal, (ii) civil, (iii) disciplinary, and (iv) administrative. With such a broad perception, liability is regulated by several laws and regulations that have evolved to stay abreast of best practices [16]. According to Al-Azri, the basic statute of Oman, promulgated by Royal Decree 101/96, largely codified the legal system in Oman [17]. In 2019, Oman adopted Royal Decree 75/2019, a new law that guides the practice and professional ethics of medicine and related health professions, bringing Omani healthcare legislation in line with international standards [18].

To improve the quality of medical practice and reduce the incidence of medical malpractice litigation, it is important to understand the patterns and characteristics of existing malpractice claims, which can provide further insight into litigious errors in clinical practice. The challenges in establishing these include the paucity of reliable data on litigation processes and there are no studies that have conducted in-depth explorations of medical litigation in Oman. To fill this gap in the literature and lay the foundations for mechanisms for the prevention and mitigation of medical errors and design evidence-based strategies to reduce litigation, the present study aimed to conduct a 12-year retrospective review investigating the characteristics of malpractice claims, the outcomes decided by the HMC, and predictors of medical errors in cases registered with the HMC in Oman.

## 2. Methodology

### 2.1 Setting

During the period between 2010 and 2021, 1284 cases raised by Omani nationals and non-Omani expatriates were registered for investigation by the Higher Medical Committee (HMC), a committee established to assess and provide technical opinions on medical errors in cases that are submitted to the judicial system, including the public prosecution, or courts, as well as the MOH. The HMC plays a crucial role in evaluating whether medical errors have occurred and offering expert insight in healthcare-related legal proceedings.

The medical specialties, the healthcare sector and the geographical locations involved in the claims were examined. Data was also collected about the referring institution for the investigation; courts, Public Prosecution, and MOH. Oman has 11 administrative provinces/governorates *(muhafazah*): Muscat, Dhofar, Musandam, Buraymi, Dakhiliyah, North Batinah, South Batinah, South Sharqiyah, North Sharqiyah, Dhahirah, and Wusta. For theoretical reasons, this study has distinguished between those living in Muscat, which is the capital of Oman, and those living in the other remaining regional settings. The urban-rural dichotomy may influence the incidence of medical lawsuits, as the quality of medical care can be influenced by systemic issues such as access to resources, the adequacy of staff and standards of training and equipment. These factors can contribute to the risk of medical errors and increase the likelihood of medical lawsuits. Thus, places of residence were conveniently classified as ‘Muscat region’ or ‘other regions’.

### 2.2 Data collection and pathways to HMC

**Fig 1:**
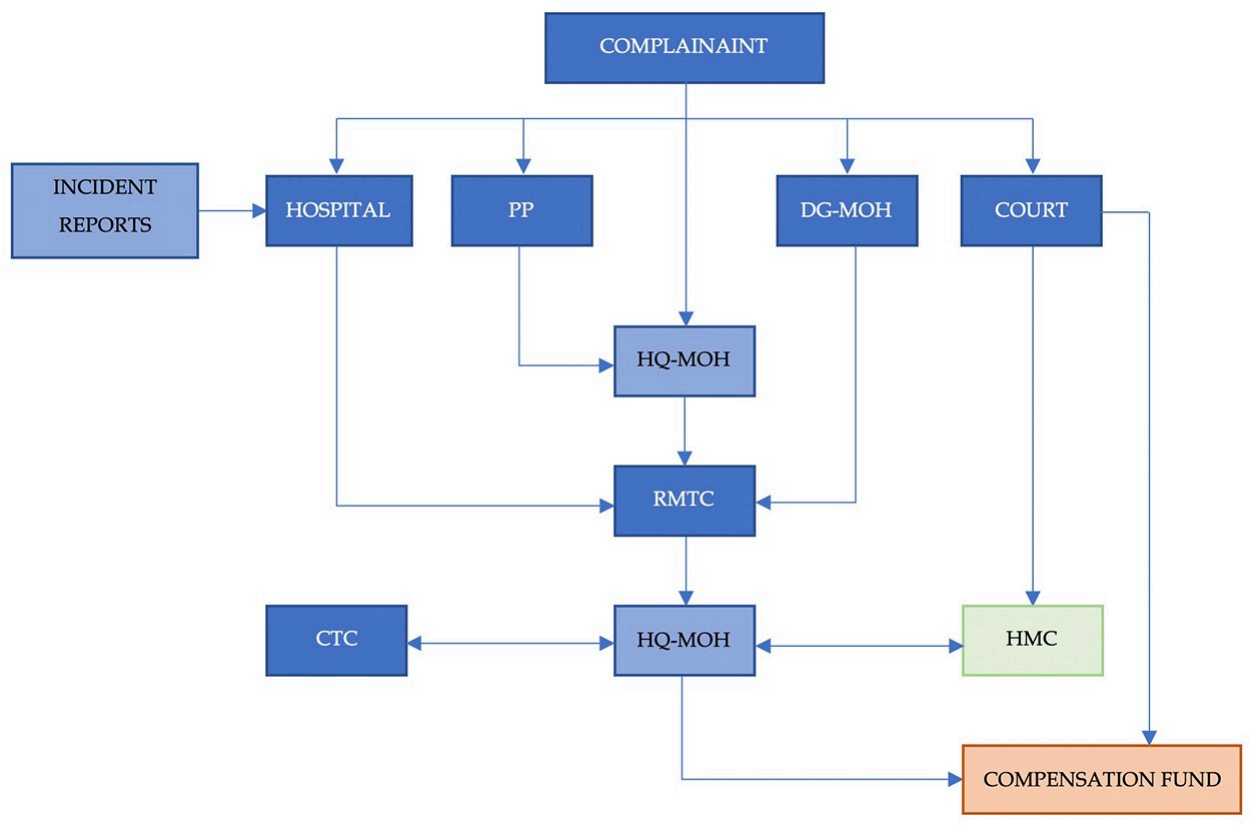
Pathways to register medical complaints in Oman. (**PP**: Public Persecutor, **DG-MOH**: Regional Directorate General of the Ministry of Health, **HQ-MOH**: The Undersecretary of Health Office at the Headquarters of Ministry of Health, **RMTC**: Regional Medical Technical Committee, **CTC**: Central Technical Committee, **HMC**: Higher Medical Committee).

The pathways to HMC are depicted graphically in Fig 1. In Oman, the handling of medical litigation has evolved and there are currently three major committees to handle these cases. First, the Regional Medical Technical Committees (RMTC) consist of senior medical professionals representing various specialties, who are allowed to seek the assistance of other senior medical professionals as Technical Experts (TE) in their investigation. RMTC investigates medical disputes or complaints raised in their region by government or private health institutes. Second, the Central Technical Committee (CTC) is similar to the RMTC, as it is also made up of a group of senior physicians who are located at the MOH headquarters, where it reviews all cases investigated by the RMTC. Following the review, the CTC then advises whether the case should be closed or if it would benefit from a referral to the HMC for further investigation. Third, those cases suspected of having medical errors regardless of the outcome of the RMTC investigation are referred to the Higher Medical Committee (HMC) for further investigation. The HMC is made up of a group of senior physicians in multiple specialties (permanent members), and the committee can invite TE’s according to the case investigation requirements to decide whether there is a medical error or harm and outline the causal relationships. Currently, in Oman, there are several ways to file a complaint if medical malpractice is suspected. The complainant(s) can file the case through various outlets within the MOH, as shown in Fig 1, where such cases are initially investigated by the RMTC. HMC directly receives all cases referred to the courts of law in case a complainant files the case through them. Additionally, HQ-MOH, which is the undersecretary of the health office at the MOH headquarters, also sends all cases suspected of medical errors to the HMC for further investigation. These cases are initially reviewed by the CTC and if they conclude that no medical error was made, then the case is closed at the MOH level and the complainant(s) are informed about the decision. However, if the complainant(s) are still not satisfied with the outcome, then they could file a case in the primary court for further HMC investigation. HMC cases registered and reported from January 1, 2010 to December 31, 2021 were reviewed and studied. In general, the HMC received 1284 complaints during this period and an investigation has been completed in 1048 of these cases, leading to the outcome of whether a medical error has occurred or not. The rest of the cases were still under investigation while this study was conducted. The data from these reports were collected by the investigators from April 1, 2021 to January 31, 2022 for further analysis.

Documented data included the specialties of the medical personnel involved, selected demographic characteristics, region of occurrence, what institution referred the case, type of medical institution involved, time between complaint registration and litigation closure, and the result of the alleged injury as ‘medical error’ or ‘no medical error’. The registered cases were further classified by specialty as follows: (1) Internal medicine (Accident and Emergency, Gastroenterology, General practitioner / Family Medicine, Hematology, General Medicine, Nephrology, Oncology, Neurology and Urology); (2) Cardiology, (3) Dermatology; (4) Surgery (General Surgery, Neurosurgery, Cardiovascular Surgery, Anesthesiology, Hand Surgery, Pediatric Surgery, Spine Surgery, and Plastic Surgery); (5) Dental; (6) Ear, Nose and Throat (ENT); (7) Obstetrics and Gynecology; (8); Orthopedics; (9) Ophthalmology; (10) Pediatrics, and (11) Others. In addition to the data mentioned above, which is required for the analysis of anonymised HMC reports, the investigators did not have access to identifiable patient data such as medical records or personal information.

### 2.3 Statistical analysis

Data were analyzed using SPSS version 25 statistical software (IBM SPSS Statistics). The study considered the results of the complete investigation of the HMC, which was classified as ‘medical error’ or ‘no error’, as a binary outcome variable. The year of registration, age, sex, nationality, region of residence of the complainants (Muscat vs. other regions), level of care provided by the health institutions involved with the complaints, number of sessions required to complete the investigation by HMC, and the waiting time to initiate the investigation were considered explanatory variables. Descriptive and inferential statistical techniques were used for data analysis. Frequency, percentage, mean, and standard deviation were used to describe the characteristics of the 1284 registered medical litigation cases, while bivariate analysis using cross-tabulation and the Chi-square test was used to analyze the differential effects of the explanatory variables on the outcome of 1048 investigated cases. Time trends in medical litigation reporting were analyzed using a line graph and the fitted simple linear regression model, assuming that the reported cases over the period follow a linear trend. To identify significant predictors of medical error, we employed multiple logistic regression analysis. A p-value <0.05 was considered statistically significant.

### 2.4 Ethical approval

The study was approved by the Research and Ethical Review and Approval Committee of the MOH of Oman (MoH/CSR/21/24217). As this is a retrospective review of HMC reports, the committee waived the need of informed consent from the patient.

## 3. Results

### 3.1 Characteristics of malpractice claims

**Fig 2:**
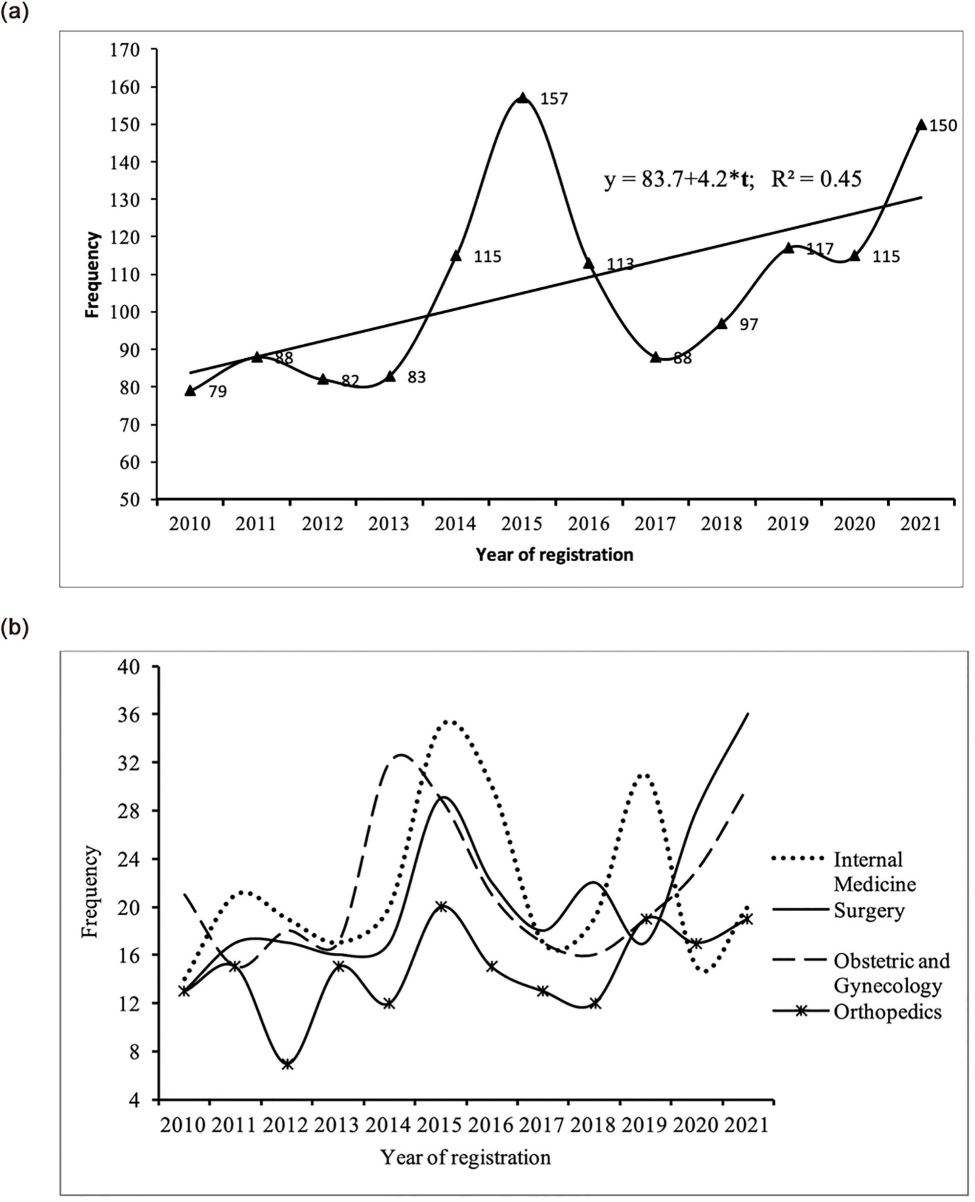
**a**: Number of cases registered in HMC by year of registration. **b**: Number of cases registered for four most commonly involved specialties in Higher Medical Committee investigations by year of registration.

**Fig 3:**
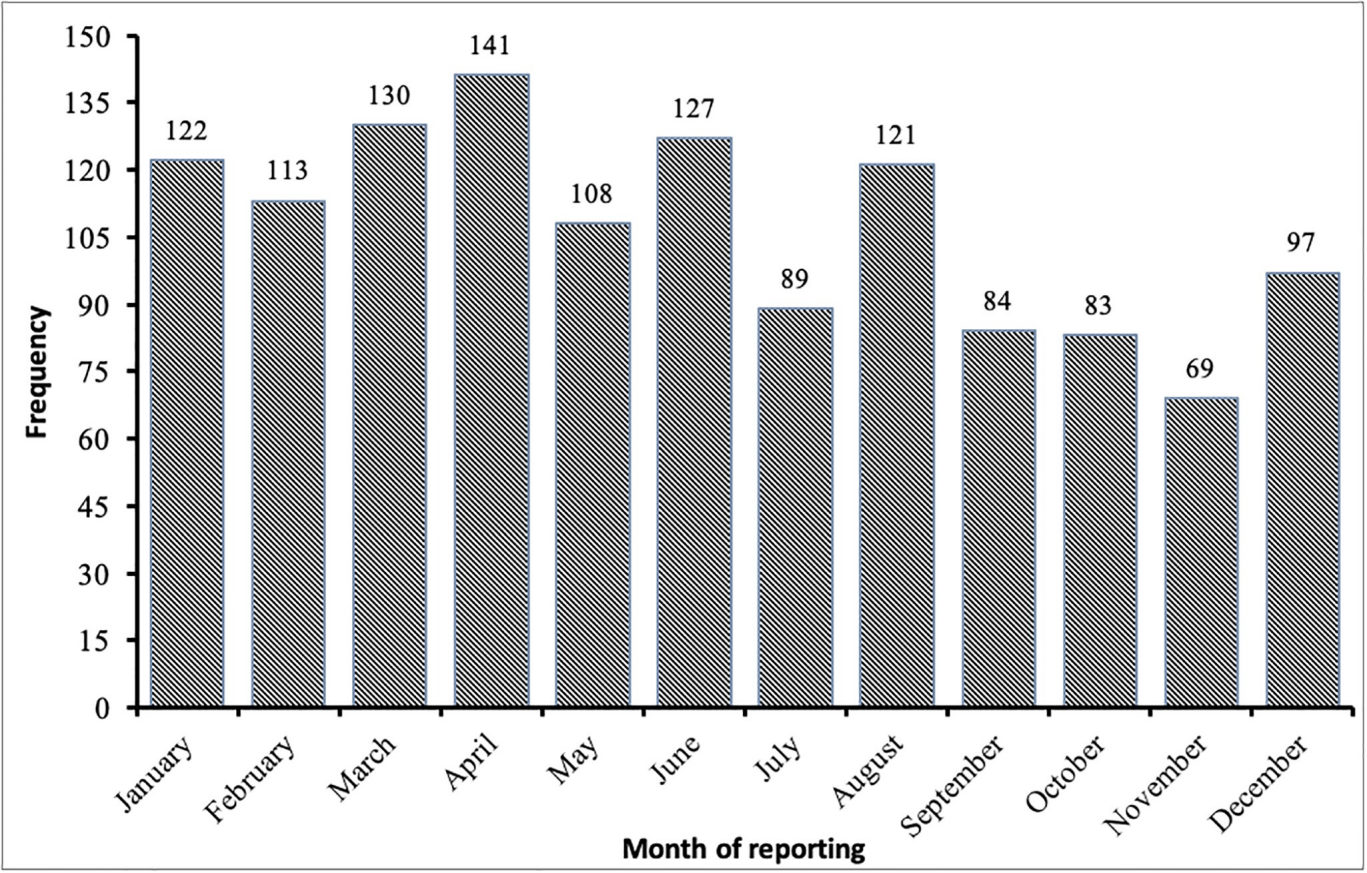
Number of cases registered in HMC by month of registration.

A total of 1284 cases were registered in the HMC between 2010 and 2020, and data from all of these cases were included in the analysis of the incidence of the registration of medical malpractice claims and the characteristics associated with the cases and complainants. Of the 1284 registered cases, 621 cases (48.4%) were referred to the HMC by the MOH, 383 (29.8%) from the court system, and 280 (21.8%) from the Public Prosecution. 1098 of the 1284 cases were registered by Omani nationals and 186 by non-Omani expatriates. The average number of cases registered with the HMC for investigation between 2010 and 2013 was 83. However, there was a significant increase in 2014 when a total of 115 cases were registered in that year, followed by 157 cases in 2015. After a decline in cases registered per year after 2015, the number of cases peaked in 2021 with 150 cases registered, as shown in Fig 2a. The average number of medical malpractice cases filed by the HMC between 2010 and 2013 is 83 cases per year, while the average number of registered cases between 2018 and 2021 is 119.75 cases per year, representing a 44% increase in caseload. The fitted trend line showed a general upward trend in cases based on medical malpractice (Fig 2a). Four of the most commonly involved specialties involved in HMC investigations were Internal Medicine, Surgery, Obstetrics and Gynecology, and Orthopedics, and Fig 2b displays the number of cases registered in each specialty by the HMC by year of registration (Fig 2b). The distribution of medical malpractice cases by reporting month indicates that there is a seasonal variation in the filing of cases for the HMC investigation in Oman (Fig 3). The rate of referral of cases to HMC for investigation varies from 5.0% in November to 11.0% in April. January to April show a higher rate of registration, which represents 39.0% of the total reported cases.

Table 1 shows the characteristics of the 1,284 HMC cases during the study period. There was almost no difference in the sex distribution of the registered cases, as half of them involved male complainants and the other half involved female complainants. The average age of the complainants was 32.0 years. The majority (70%) of the registered cases were received from adult complainants between 18 and 59 years of age, and approximately half of the cases (51%) were complainants between 18 and 39 years of age. About 86% of the litigations were brought by Omani complainants compared to 14.0% of non-Omani complainants.

**Table 1:** Percentage distribution of registered medical malpractice cases by selected background characteristics, Oman 2010–2021.

Characteristics	Frequency	Percentage
**Total**	1284	100.0
**Sex**		
Male	640	49.8
Female	644	50.2
**Age group (in years)**		
<1	67	5.4
1–4	67	5.4
5–17	123	10.0
18–39	632	51.2
40–59	228	18.5
60+	117	9.5
Mean age (SD)	31.8 (19.1)	
**Nationality**		
Omani	1098	85.5
Non-Omani	186	14.5
**Region**		
Muscat (urban)	549	42.8
Other Regions	735	57.2
Referring Institution to HMC***		
Ministry of Health	621	48.4
Court System	383	29.8
Public Prosecutor	280	21.8
**Type of health institution involved**		
Ministry of Health	878	68.4
Other governmental institutions not related to the Ministry of Health	63	4.9
Private	343	26.7
**Specialties**		
Internal Medicine	258	20.1
Dermatology	21	1.6
Dental	79	6.2
ENT	31	2.4
Surgery	252	19.6
Obstetrics and Gynecology	258	20.1
Ophthalmology	60	4.7
Orthopedics	177	13.8
Pediatrics	110	8.6
Others	38	3.0

Note: **HMC**: Higher Medical Committee

Slightly less than half (48.4%) of the registered cases were received through the MOH. The cases received from the Primary Courts (PC) represent 29.8% of the total cases, and those received from the Public Prosecution represent 21.8%. It should be noted that more than two-thirds (68.4%) of medical malpractice lawsuits were brought against public hospitals/MOH, while 26.7% of cases were brought against the private health sector, as shown in Table 1.

The locations of the health institutions involved in the litigations showed that the currently defined urban region (Muscat region) generated 42.8% of the complaints. The remaining 57.2% of the cases occurred in the other 10 collective regions.

The distribution of medical litigations in different medical specialties showed that obstetrics and gynecology was the specialty most frequently involved in the cases raised (20.1%), closely followed by internal medicine and associated subspecialties (19.7%), surgery (17.6%), and orthopedics (13.8%). Medical malpractice litigation claims were found to be low for the specialties of dermatology (1.6%), cardiology (2.4%), ENT (2.4%), and ophthalmology (4.7%).

### 3.2 The outcome of HMC investigations

**Fig 4:**
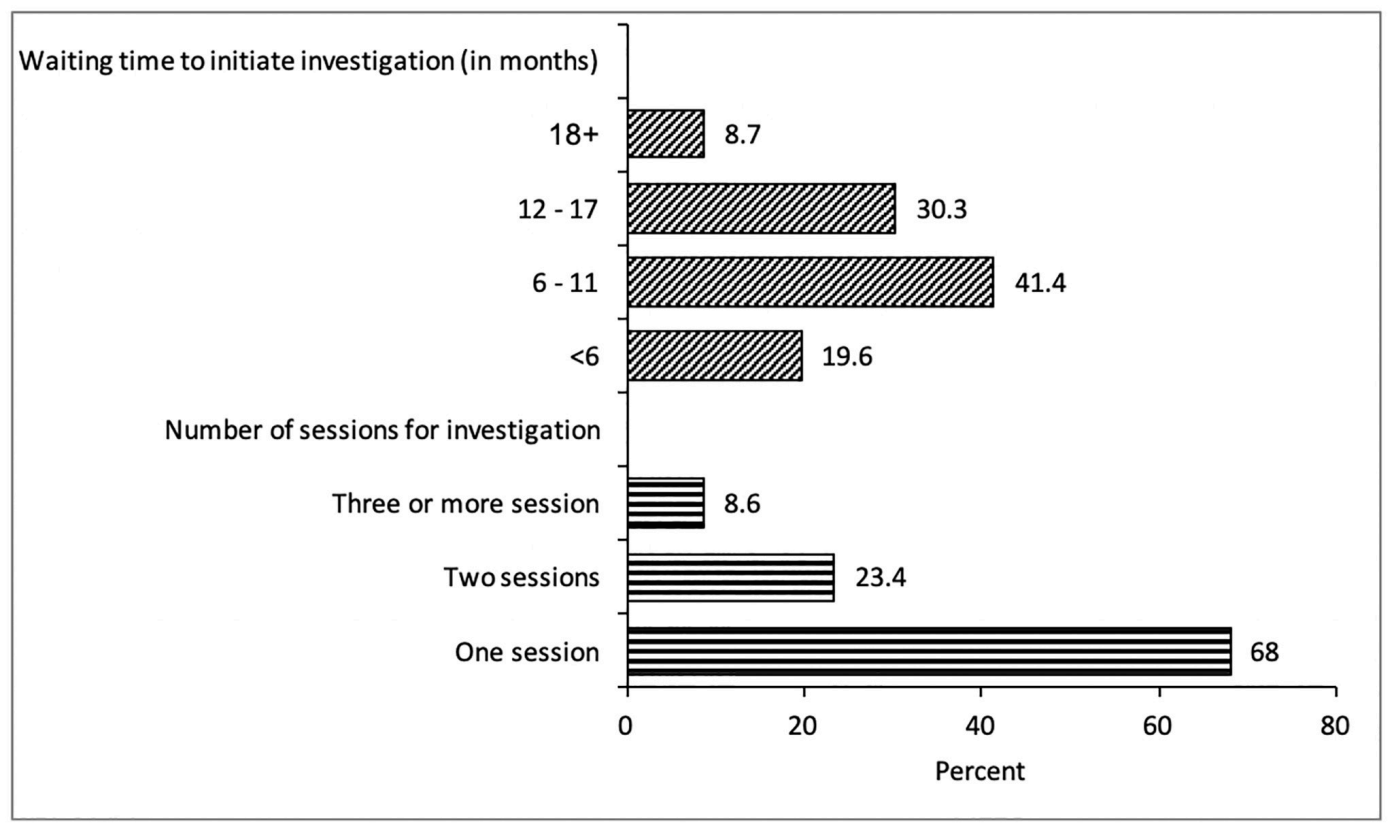
Percentage distribution of completed investigations by number of investigations and waiting time to initiate investigation by HMC.

The 1048 cases of litigation with HMC investigations completed were analyzed to assess potentially significant background characteristics associated with a final outcome of ‘medical error’ or ‘no medical error’. Of the 1048 cases, 495 cases were referred to the HMC by the MOH, 306 from the court system and 247 from the Public Prosecution. More than two-thirds (68.0%) of the HMC-investigated cases were completed within one investigative session, while 23.4% of the cases were investigated in two sessions, and 6.7% needed three or more investigative sessions, as shown in Fig 4. The average waiting time to initiate the HMC investigation was 10.2 months. The waiting time was less than 6 months for about a fifth (19.6%) of the investigated cases, while it was 6–11 months for 41% of the cases, and it took 18 months or more for about 9% of the cases (Fig 4).

The HMC investigation found no medical errors in almost 51% of the cases. Table 2 presents the differentials of medical error in the selected characteristics with a p-value for the Chi-square test. Age, sex, region, and waiting time to initiate the HMC investigation did not show significant differences in the rate of observed medical errors. Medical error was found to be more prevalent among non-Omani nationals than among Omani nationals (58.6 vs. 47.9%). The error rate was found to be higher in the urban region than in other regions (52% vs. 48%). The rate of medical error increased with the number of sessions required to complete the HMC investigation. The error rate was also found to be higher among the MOH-referred cases (56.4%) and lower among the court system-referred cases (35.3%). The error rate was higher for cases related to private hospitals rather than those of public hospitals under the MOH (57.6% vs. 47.2%) (Table 2).

**Table 2:** HMC investigation results (medical error/no medical error) according to background characteristics, Oman 2010–2021.

Characteristics	Outcome of the HMC investigation, N = 1048			
	% of Medical Error N = 518	% of No Medical Error N = 530	Total	Frequency
**Total**	49.4	50.6	100.0	1048
**Number of sessions required to complete the investigation** ***				
One session	44.5	55.5	100.0	713
Two sessions	58.4	41.6	100.0	245
Three or more sessions	68.6	31.4	100.0	90
**Waiting period (in months)**				
<6	55.1	44.9	100.0	205
6–11	50.5	49.5	100.0	434
12–17	46.2	53.8	100.0	318
18+	42.9	57.1	100.0	91
**Mean waiting time (SD)**	9.7 (4.8)	10.6 (4.9)		
**Sex**				
Male	46.8	53.2	100.0	513
Female	52.0	48.0	100.0	535
**Age group (in years)**				
<1	50.0	50.0	100.0	64
1–4	46.0	54.0	100.0	50
5–17	56.9	43.1	100.0	102
18–39	49.2	50.8	100.0	522
40–59	51.1	48.9	100.0	190
60+	45.4	54.6	100.0	120
Mean age (SD)	31.3(18.7)	32.0(19.6)		
**Nationality** **				
Omani	47.9	52.1	100.0	896
Non-Omani	58.6	41.4	100.0	152
**Region**				
Muscat (urban)	52.0	48.0	100.0	425
Other Regions	47.7	52.3	100.0	623
**Referring Institution to HMC** ***				
Ministry of Health	56.4	43.6	100.0	495
Court System	35.3	64.7	100.0	306
Public Prosecutor	53.0	47.0	100.0	247
**Type of health institution involved** **				
Ministry of Health	47.2	52.8	100.0	729
Other governmental institutions not related to the Ministry of Health	40.0	60.0	100.0	55
Private	57.6	42.4	100.0	264
**Specialty** **				
Internal Medicine	43.9	56.1	100.0	221
Dermatology	76.9	23.1	100.0	13
Dental	64.8	35.2	100.0	54
ENT	69.2	30.8	100.0	26
Surgery	53.0	47.0	100.0	202
Obstetrics and Gynecology	49.5	50.5	100.0	216
Ophthalmology	44.6	55.4	100.0	56
Orthopedics	42.7	57.3	100.0	143
Pediatrics	49.5	50.5	100.0	91
Others	50.0	50.0	100.0	26

Note: sig. p<0.05;

** p<0.05,

*** p<0.001;

**HMC**: Higher Medical Committee

Although the dermatology specialty had the lowest number of cases registered with them, the conclusions of the HMC investigations indicated that the highest error rate (76.9%) of errors was associated with the dermatology specialty. However, medical errors were found to have occurred in only about 50% of the cases involving the obstetrics and gynecology specialty, although the highest number of cases were filed against this specialty. The rate of medical errors was found to be the lowest among cases involving cardiology specialty.

### 3.3 Predictors of medical errors

The results of the multiple logistic regression analysis of medical errors, as presented in Table 3, identified nationality (i.e. Omani vs. non-Omani), the institution that referred the litigation cases to HMC, the type of health institutions involved with the cases, the number of investigative sessions, and waiting time to initiate the HMC investigation as significant predictors of medical errors. The odds of medical error were found to be 42% lower among Omani nationals compared to their non-Omani expatriate counterparts (AOR = 0.581, 95% CI: 0.387–0.874). The odds of medical error were 65% lower among the cases referred to the HMC by the court system than among the cases referred by the Public Prosecutor or the MOH (AOR = 0.349, 95% CI 0.236–0.518). The risk of medical error was found to be approximately 1.5 times higher among cases related to private health institutions than among cases related to public health institutions under the MOH (AOR = 1.444, 95% CI: 1.017–2.168). The number of sessions required to complete the HMC investigations showed a significant positive association with medical errors, as the odds of medical errors increased with the number of sessions. The risk of medical errors was found to be almost four times higher among the cases for which the investigation was completed in three or more sessions (AOR = 3.993, 95% CI: 2.233–7.137). The waiting time to initiate the HMC investigation was found to have a negative association with medical errors. For example, the risk of medical errors was 1.8 times higher in cases with a waiting time of less than 6 months compared to cases with a waiting time of 18 months or more (AOR = 1.772, 95% CI: 1.026–3.062). The specialty involved appeared to be a significant predictor of medical errors. Compared to internal medicine, specialties such as dermatology (AOR = 4.580, 95% CI: 1.149–18.249), ENT (AOR = 3.629, 95% CI: 1.407–9.357), dental (AOR = 2.091, (5% CI: 1.045–4.185) and surgery (AOR = 1.692, 95% CI: 1.090–2.626) had significantly higher odds of medical errors.

**Table 3:** Factors associated with medical errors in registered medical malpractice cases, Oman 2010–2021.

Characteristics	Adjusted Odds Ratio (AOR)	95% CI of AOR	p-value
**Sex**			
Male	0.786	0.578–1.067	0.123
Female (ref.)	1.000		
**Age group (in years)**			
<1 (ref.)	1.000		
1–4	0.831	0.373–1.854	0.651
5–17	1.343	0.644–2.798	0.431
18–39	1.241	0.622–2.475	0.540
40–59	1.181	0.565–2.468	0.658
60+	1.102	0.497–2.446	0.811
**Nationality** **			
Omani	0.586	0.392–0.875	0.009
Non-Omani	1.000		
**Region**			
Muscat (urban)	0.938	0.696–1.264	0.673
Other Regions	1.000		
**Referring Institution to HMC** **			
Ministry of Health	1.089	0.785–1.512	0.609
Court System	0.364	0.248–0.533	<0.001
Public Prosecutor (ref.)	1.000		
**Type of health institution involved** **			
Ministry of Health (ref.)	1.000		
Other government institution not related to the Ministry of Health	0.872	0.465–1.635	0.104
Private	1.476	1.015–2.148	0.042
**Number of sessions required to complete the investigation** ***			
One session (ref.)	1.000		
Two sessions	2.020	1.474–2.768	<0.001
Three or more sessions	3.576	2.051–6.235	<0.001
**Waiting period (in months)**			
<6	1.694	1.061–2.908	0.046
6–11	1.432	0.873–2.350	0.155
12–17	1.167	0.704–1.934	0.550
18+ (ref.)	1.000		
**Specialties**			
Internal Medicine (ref.)	1.000		
Dermatology	4.966	1.258–13.607	0.022
Dental	2.193	1.098–4.377	0.026
ENT	4.098	1.599–9.507	0.003
Surgery	1.973	1.287–3.026	0.002
Obstetrics and Gynecology	1.166	0.744–1.828	0.502
Ophthalmology	1.550	0.811–2.960	0.184
Orthopedics	1.341	0.834–2.157	0.226
Pediatrics	1.816	0.920–3.585	0.085
Others	1.519	0.633–3.642	0.349

Note: ref. = reference category with odds ratio 1.000; sig. p<0.05;

** p<0.05,

*** p<0.001;

**HMC**: Higher Medical Committee

## 4. Discussion

The prevalence of medical errors varies widely throughout the world and is difficult to accurately quantify due to a lack of standardisation in reporting and defining what constitutes a medical error [19]. Despite the lack of taxonomy of medical errors, there is evidence to suggest that they are a significant and widespread problem that affects millions of people every year. In the United States, the Institute of Medicine Committee on Quality of Health Care estimated that medical errors cause between 44,000 and 98,000 deaths each year [20]. More recent estimates suggest that the number of deaths due to medical errors may be even higher, with one study putting the figure at 251,000 deaths per year [21]. In the United Kingdom, the National Health Service estimated that there are approximately 250,000 adverse events (including medical errors) in the National Health Service each year, causing harm to about 100,000 patients [22]. In Australia, a 2016 study estimated that medical errors can cause up to 17,000 deaths each year [23]. The prevalence of lawsuits against healthcare workers in Oman and its neighboring Gulf Cooperation Council (GCC) countries has received little attention. In recent years, it has been widely recognised that the healthcare sector in GCC countries has been rapidly expanding and modernising, and this growth has been correlated with the increase in the number of medical malpractice cases. However, the legal framework for medical malpractice in the GCC countries is still evolving, and the procedures for filing and settling lawsuits can be complex. With this background, this study was conducted to describe the characteristics of malpractice claims, the results of the medical liability committee, and the predictors of medical errors in Oman.

### 4.1 Characteristics of malpractice targets

Morbidity and mortality are significantly affected by medical errors. Medical errors account for 10% of all deaths in the United States, making them the third leading cause of death, although there are differing opinions regarding this statistic [24, 25]. However, medical mistakes can have major consequences, and patient safety policies should be improved to mitigate and protect against them. The number of malpractice litigation cases in Oman filed in the HMC fluctuated during the study period, with a total of 1284 cases registered between 2010 and 2021. The number of registered cases peaked twice, in 2015 and 2021. In 2015, there were changes to the internal referral system from MOH to HMC, which is considered the main cause of the surge in cases during that year. Following this, a short decreasing trend was observed until 2019, leading to another surge in registered cases that peaked in 2021. This second surge was related to the reduced functioning of the HMC during the onset of the COVID-19 pandemic, leading to a backlog of cases. However, there has been an overall increase in the filing of medical malpractice cases in Oman during the study period compared to previous years, with an increase of 31% that mirrors international trends [26, 27]. The increase could be related to multiple factors, such as the growth of the Omani population, the increase in the number of medical facilities, healthcare providers, and the complexity of medical and surgical interventions. Increased litigation could also be attributed to increased awareness within the community of the standards of medical care, medical errors, and the rights of patients to complain and litigate against healthcare professionals [28]. Unlike previous generations, Oman now has access to high levels of Internet connectivity, and social networks have allowed the discussion of topics that were once hidden or discouraged [29, 30]. Oman has generally triumphed over the traditional enemy of health in developing countries, namely communicable diseases. However, a consequence of the recent rapid socio-economic changes in Oman is the emergence of non-communicable diseases. Thus, according to Al-Mandhari et al [31], ‘the existing model of health services in the country, top-down, professionally driven and cure-oriented, is increasingly unable to deal with this new assortment of health problems” (pp. 319). It remains to be established whether the rising tide of non-communicable diseases has caused dissatisfaction with healthcare services in Oman, which in turn could fuel litigation for alleged medical malpractice. It is worth noting that, while there is an increase in medical litigation in Oman, the trend captured in the present study appears to be low compared to international statistics, although such a conclusion may be premature since the cited comparison study is based on primary care rather than general national healthcare trends [32]. However, several factors may contribute to the lower rate of medical litigation in Arab countries, including limited access to legal services, lack of regulatory frameworks, and cultural attitudes that discourage patients from questioning healthcare providers’ decisions or holding them responsible for adverse outcomes [33]. Therefore, more studies are warranted in these regions to lay the groundwork for culturally sensitive strategies to increase patients’ awareness of their rights.

In terms of demographics of malpractice claims, the present cases were evenly distributed between male and female complainants. The majority of cases (67%) were raised by adult complainants aged 18–60 years, and almost half of them were 19–40 years with an average age of 32 years. This age group represents the structure of the Oman population, where most of the population is under 35 years of age [10]. In terms of nationality, the majority of disputes (86%) were brought up by Omani complainants. This is not a surprising finding when considering that almost 95% of all visits to MOH health institutions are by Omanis [34]. It should be noted that nearly 39% of the population of Oman is made up of expatriate workers from different parts of the world [35].

The MOH received slightly less than half (48.4%) of the registered cases, while the Primary Courts (PC) received 28.5% and the Public Prosecution received 21.8%. More than half of medical malpractice lawsuits were filed against government MOH hospitals (68.4%), while 26.7% were brought against the private health sector. The MOH provides healthcare services to most of the population of Oman, with more than 51 MOH hospitals compared to the 4 other non-MOH government hospitals and the 27 private hospitals [34]. Complex cases are generally not handled by private healthcare institutions and, in general, are referred to MOH or tertiary level hospitals due to limited facilities.

The urban region (Muscat) represented 42.8% of the complaints, with the rest of the individual governorates receiving fewer complaints. This implies that the presently defined urban region, i.e. Muscat alone, bore the burden of the highest number of litigations than any other individual region. This may be explained by the high population density and the increasing number of government and private healthcare facilities concentrated in Muscat. This includes several tertiary care centers, which generally deal with complex cases that require higher levels of interventions that carry a higher risk of complications or adverse outcomes. The lowest number of litigations was received from those regions where the population density is the lowest in Oman.

Most of the lawsuits were brought against surgical specialties (obstetrics and gynecology, orthopedics, and general surgery). In the United States, Jena et al analysed the 1992–2005 misconduct data of all physicians covered by professional liability insurance companies (n = 40,916) based on 25 specialty fields [36]. The percentage of physicians who encounter a claim every year varied between different medical specialties. The percentage ranged from 19.1% for neurosurgery, 18.9% for pulmonary and cardiovascular surgery, 15.3% for general surgery, 5.2% for family medicine, 3.1% for children and 2.6% for psychiatry. In Italy, Bolcato et al assessed medical professional liability in tertiary hospitals from 2003 to 2019 [37]. The claims were classified as follows: 37% were related to the surgical field, 17% were related to internal medicine, and 35% were related to emergency care. In terms of types of medical errors, compensation was granted in 30% of cases involving diagnostic errors, 26% of cases involving therapeutic errors, 47% of cases involving execution errors, and 55% of cases involving organizational deficiencies. In general, studies from different populations suggest that surgical specialties (obstetrics and gynecology, orthopedics, and general surgery) appear to be more liable for a malpractice claim or lawsuit [26, 38, 39]. This is consistent with the findings of the present study. This could be explained by the high degree and level of intervention and the need for immediate and prompt decisions and actions in these specialties, which contribute to a higher level of stress and a lower quality of care and the doctor-patient relationship. Stress and burnout have been established to be associated with medical errors [15, 40]. Stress and high workloads could also lead to poor quality medical records, which are important for after events and medical cases. Documentation of counseling sessions, informed consent forms, and other documentation are important evidence in the investigation. It is recommended to mention all potential complications with informed consent, not only to protect the physician but also to emotionally prepare the patient in the event of a complication. Therefore, such important pre-intervention counseling and consent are critical, as the patient might misinterpret the intervention complication(s) as a medical error instead [41].

### 4.2 Outcomes of the medical liability committee

The HMC completed investigations for a total of 1048 cases during the study period, of which almost half of the cases did not have medical errors. The waiting time to initiate the investigation for the cases referred to HMC was variable, ranging from less than six months to more than 18 months of waiting time from the time of case registration. An explanation for the longer duration may be partially related to the frequency of HMC meetings in the initial period of the study (before 2015), when it used to take longer to finalize the cases. Additionally, the logistic issue of getting complete medical records from all hospitals involved in the case used to take longer. However, this waiting period has decreased in recent years, as the HMC has undergone internal arrangements to speed up the process of investigating and finalising their reports within three months, as mandated by a new law in the country that came into effect in 2019 unless the HMC requests an extension. At the time of case registration, the HMC prioritises the investigation process depending on the nature of the case referred, including the institution that referred the case. For example, cases referred from the court and Public Prosecution get the top priority, followed by those referred from MOH. Additionally, cases with high suspicion of medical error, especially when death is involved, are prioritised for expedited HMC investigation.

When non-Omani nationality complainants filed a medicolegal case, the chances of confirming that the error occurred were greater than among those of Omani nationals. One possible explanation may be differences in how non-Omani perceive medical errors compared to nationals. The threshold for raising complaints may be lower among the Omani nationals, who likely have less reason to be cautious about the lengthy and tedious process compared to non-nationals. Furthermore, language barriers between patients and healthcare professionals have been found to lead to miscommunications, as well as to decrease the quality of healthcare and patient satisfaction. This may have been a possible contributing factor in cases of medical error registered by expatriate patients who were not fluent in English or Arabic [42].

During the investigation process, the HMC conducts investigative sessions to review medical records, meet the complainant(s), and interview the involved staff in the presence of HMC members and TE’s. Each session can last up to four hours. The investigation was carried out in one investigative session in 68% of the cases. The more HMC sessions that occurred, the higher the probability of confirming errors. This could be due to the complexity of the case and the involvement of more staff, as well as the HMC’s conscientiousness during the investigation to reach an accurate final decision. Other reasons for conducting more than one investigative session vary between cases, such as missing medical records, the presence of new staff involved in the investigation, or the identification of new information that skewed the direction of the investigation. This process can be expedited by applying an electronic system that links national electronic health records with HMC.

Cases referred to the MOH and public prosecutors have a higher chance of medical error than cases referred to the court. The cases referred to the court are not filtered by the local technical committee compared to those referred from MOH and Public Prosecution, which go through the RMTC and CTC and therefore have a higher potential for medical error. Similar observations were made for cases referred to private sector hospitals, which leads to the conclusion that they bear a higher burden of medical errors compared to MOH hospitals.

The frequency of medical malpractice cases in the dermatology specialty was low. However, the results of the HMC investigation found the highest medical error rate among these cases. Most medicolegal litigation in dermatology was raised due to the negative outcome of aesthetic surgery and procedures, mainly in private practice. However, despite the highest rate of complaints against obstetric and gynecological services, only 50% of the cases investigated have confirmed the presence of medical malpractice. Finally, the number of cases related to the cardiology specialty was relatively low and the confirmation of medical errors in these cases was also less likely.

To date, this is the first study of its kind to emerge from emerging economies of the GCC. The present data suggest that several factors can predict medical errors, including the complainant’s nationality (i.e. Omani vs. non-Omani), the referring institution, the type of health institution involved, the specialty in question, the number of HMC investigation sessions, and the waiting time for the investigation to be initiated. A negative correlation was found between the waiting time to complete the investigation and the HMC outcome of the medical error. This analysis also indicates some areas that would require vigilance to reduce court cases. Dermatology, dental, and surgical specialties have a higher risk of concluding medical errors than internal medicine. This implies that specialties that involve regular intervention tend to accrue higher litigation rates than other specialties such as internal medicine and its associated specialties.

Malpractice claims analysis is a commonly employed method to identify areas for improvement in patient safety and quality improvement. In general, the study findings provide information that can guide healthcare institutions, policymakers, and professionals in implementing preventive measures. Addressing the identified factors associated with malpractice claims could be used to prevent medical errors and minimize the burden of medical malpractice litigation.

### 4.3 Limitations

Although this study on medical malpractice litigation in Oman provides valuable information, it is important to consider some limitations that can affect the interpretation of the findings. *First*, the present study is retrospective and relies on existing data that may not have been collected with the specific research question in mind. Similarly, retrospective studies are observational and therefore cannot establish definitive causality between variables.

*Second*, the number of cases investigated by the HMC does not represent all medical litigations or complaints about medical errors in Oman. There were complaints investigated by the RMTC that were not referred to the HMC as the cases were either deemed to have no medical error at the RMTC level or for which the complainant(s) did not wish to pursue the claim further. Related to this, the study relies on the availability of documented medical malpractice cases. It is possible that not all cases were officially reported and documented, which in turn may have potentially led to reporting bias. Cases with less severe consequences or those settled outside of the legal system may be under-represented. The results presented here are only for the registered cases for which the HMC completed its investigation during the study period (82% of the cases), and therefore 18% of the registered cases that are currently under investigation were not included in the analysis of the results.

*Third*, the present study focuses on cases of medical malpractice registered with the Higher Medical Committee (HMC) in Oman over a 12-year period. Findings may not be generalizable to other regions or countries with different health systems, legal frameworks, or cultural contexts. Therefore, caution is needed when extrapolating the results beyond Oman.

*Fourth*, in the present analysis, seasonality appears to have had an impact on registered cases. During the summer months, people may have chosen to travel abroad for medical care or for personal reasons. This could have resulted in a decrease in healthcare utilization and fewer visits to health facilities. As a result, medical errors that did occur could be under-reported or unnoticed if individuals delayed seeking medical attention or received care outside of Oman. This would require further scrutiny, since this could be an artefact of data collection.

*Finally*, medical recordkeeping and litigation are nascent in the country. In fact, data cleaning was essential in the present analysis to remove what was deemed spurious outliers. In addition to being limited by being a retrospective study, to increase accountability in healthcare in Oman, a more vigilant mechanism is needed to track the typology and outcome of medical litigation. The question remains whether the current observed trend could be the tip of the iceberg. Related to this, the study identifies certain predictors of medical errors, such as the complainant’s nationality and the type of health institution involved. However, it is important to recognise that there may be additional confounding factors not considered in the analysis that could influence the results. These factors could include socioeconomic status, educational level, or healthcare access disparities.

Due to the aforementioned limitation, it is worth conducting a longitudinal analysis to track trends and patterns of medical malpractice litigation in Oman beyond the 12 years covered in the study. This would discern whether the observed upsurge in recent years persistently escalates, stabilizes or recedes, as well as aid in identifying any trends over time. Comparative studies conducted between Oman and other GCC countries are also warranted, since the regions have similar health systems and cultural contexts. Such studies can provide information on similarities and differences in trends, outcomes, and predictors of medical malpractice, allowing cross-learning and identification of best practices. Finally, if the current trend observed would appear to be valid, then the contributing factors to medical errors and the ethical and legal dimensions of medical malpractice could be explored with qualitative research, such as through interviews or focus groups with patients, healthcare workers, health policy professionals, and legal experts. This would provide rich insight into experiences, perspectives, and contextual factors that may not be captured by a quantitative analysis alone.

## 5. Conclusion

The present study examined medical malpractice litigation in Oman from 2010 to 2021, analyzing data accrued from HMC cases registered with the HMC for which the investigation had been completed according to the existing system. Most of the litigations were initiated by adult Omani complainants, were predominantly from the urban Muscat region, and were frequently related to public hospitals. The most common specialties involved in litigation were obstetrics and gynecology, internal medicine, surgery, and orthopedics. About half of appeals or grievances were dismissed due to lack of medical negligence or malpractice. The predictors of medical errors included nationality (i.e., Omani vs. non-Omani), the institution that referred the case, the specialty and type of health institution involved, the number of investigation sessions required and the waiting time for the start of the HMC investigation. Some of the associated factors, pending further scrutiny, have the potential to be utilised to design preventive measures against medical errors.

An upward trend in the incidence of medical malpractice litigation was noted, however, the present research is marred by many potential confounders. More research with a more robust methodology is required to indicate the accuracy of this trend and to shed light on the contributing factors to medical malpractice and errors in Oman. The results of the present study are necessary to lay the groundwork for contemplating mechanisms to minimize the rates of medico-legal cases in Oman, as well as to increase literacy on medical errors amongst the public and healthcare workers.

## Supporting information

S1 Checklist STROBE statement—Checklist of items that should be included in reports of observational studies. (DOCX)
